# Multi‐regional Organoid Biobank Reveals FAK‐ACSL1‐Driven Doxorubicin‐Resistance and Predictive Biomarkers in Breast Cancer

**DOI:** 10.1002/advs.76541

**Published:** 2026-07-20

**Authors:** Hao Xu, Zhijun Qin, Jiapeng Yang, Baoyuan Zhang, Manqing Cao, Peng Wang, Zaiqi Wang, Jinghui Cheng, Lei Wang, Hong Liu, Hui Yang

**Affiliations:** ^1^ The Second Surgical Department of Breast Cancer Tianjin Medical University Cancer Institute & Hospital, National Clinical Research Center for Cancer Tianjin P. R. China; ^2^ Tianjin’ s Clinical Research Center for Cancer Tianjin Medical University Tianjin P. R. China; ^3^ Key Laboratory of Breast Cancer Prevention and Therapy Ministry of Education Tianjin Medical University Tianjin P. R. China; ^4^ Key Laboratory of Cancer Prevention and Therapy Tianjin P. R. China; ^5^ Department of Breast Surgery Puyang Oilfield General Hospital Affiliated with Xinxiang Medical College Puyang Henan P. R. China; ^6^ Translational Cancer Research Center Peking University First Hospital Beijing P. R. China; ^7^ InxMed (Shanghai) Co. Ltd Shanghai P. R. China; ^8^ Department of Thoracic Surgery The Fourth Hospital of Hebei Medical University Shijiazhuang Hebei P. R. China; ^9^ State Key Laboratory of Natural and Biomimetic Drugs Peking University Beijing P. R. China

**Keywords:** breast cancer, drug combination therapy, drug resistance mechanism, FAK signaling, organoid

## Abstract

Inter‐tumor and intra‐tumor heterogeneity present significant challenges to achieving precise treatment in breast cancer (BC). To address this, we established a living biobank comprising 68 patient‐derived organoids (PDOs) from 50 patients across various regions, accurately maintaining the histological, genomic, and transcriptomic characteristics of the original tumors. Paired profiling demonstrated high concordance in mutation burden, copy number alterations, and molecular subtypes, capturing both inter‐ and intra‐tumor heterogeneity. Drug screening with four clinically used agents revealed pronounced response variability, enabling the development of multi‐gene expression signatures predictive of drug sensitivity. ACSL1 emerged as a key mediator of Doxorubicin resistance, enriched in malignant epithelial cells exhibiting focal adhesion/MAPK pathway activation and immune‐evasive features. Functional and mechanistic analyses identified focal adhesion kinase (FAK) as an upstream regulator of ACSL1, mediating resistance via ERK and STAT3 signaling. Inhibiting FAK or ACSL1 through pharmacological methods increased the sensitivity of resistant PDOs to Doxorubicin. The combined treatment was effective in decreasing tumor growth and preventing metastasis in xenograft models. Clinically, elevated ACSL1 expression correlated with advanced disease and poor survival. Collectively, these findings define BC heterogeneity, establish prognostic biomarkers, as well as uncover a means of Doxorubicin resistance that supports rational combination therapy.

## Graphical abstract

This study established a high‐quality organoid biobank derived from 68 tumor sites across 50 Chinese patients, elucidated the drug sensitivity‐based molecular subtyping in breast cancer, and revealed a novel mechanism of drug resistance mediated by the FAK‐ACSL1 pathway. Targeting the FAK‐ACSL1 pathway in breast cancer is expected to yield enhanced antitumor efficacy.

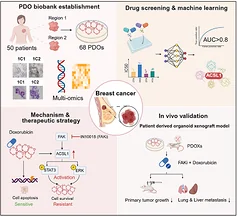

## 1 Introduction

Breast cancer (BC) is a major malignancy and the leading cause of cancer‐related death among women worldwide [1]. Despite substantial advances in diagnostics and therapeutics, persistent drug resistance remains a major barrier to durable clinical responses [2, 3, 4]. This resistance, driven by intrinsic tumor heterogeneity and the tumor microenvironment, directly contributes to treatment failure, disease recurrence, progression, and reduced survival.

BC displays pronounced heterogeneity across established molecular subtypes, which underlies its variable clinical behavior and therapeutic responses [5, 6, 7]. However, these subtypes lack sufficient resolution to reliably predict individual drug sensitivity or resistance, underscoring the need for experimental models that more accurately capture patient‐specific tumor biology to advance precision oncology.

Overcoming drug resistance requires a mechanistic understanding of its diverse drivers [8, 9], including upregulation of drug efflux transporters, enhanced DNA damage repair, dysregulated apoptotic signaling, dynamic interactions within the tumor microenvironment (TME) [10], persistence of cancer stem cells [11, 12, 13], and epigenetic reprogramming [2, 14]. FAK is a non‐receptor tyrosine kinase and it regulates extracellular matrix (ECM) pathway through integrins and growth factor receptors [15]. Recent studies highlight FAK inhibition (FAKi) as a promising strategy to reverse platinum resistance in ovarian cancer [16] and to enhance immunotherapy efficacy in pancreatic ductal adenocarcinoma (PDAC) [17].

Patient‐derived organoids (PDOs) serve as sophisticated 3D models that accurately mirror the histological, molecular, genetic, and tumor microenvironment features of individual patients [18, 19, 20, 21, 22]. This high fidelity makes PDOs powerful platforms for dissecting drug resistance mechanisms and performing clinically predictive drug screening. However, previous breast cancer organoid studies were limited by insufficient multi‐region sampling, precluding systematic interrogation of genomic and functional intratumor heterogeneity (ITH). Generating a large‐scale, multi‐region living biobank therefore supporting tumor heterogeneity analysis, prognostic biomarkers development for patient differentiation, and of resistance elucidation to guide new treatment strategies.

This study entailed establishing a live biobank composed of 68 PDOs sourced from 50 breast cancer patients across different cases. Utilizing this resource, we analyzed the genetic and phenotypic diversity, performed drug screenings relevant to clinical outcomes with corresponding patient responses, and identified molecular biomarkers that can predict treatment responses, and uncovered a Doxorubicin resistance mechanism that guided the development of a synergistic combination therapy linking Doxorubicin with FAK inhibition.

## 2 Results

### 2.1 A Biobank of Organoids Spanning Multiple Regions Accurately Mimics the Tissue and Multi‐Omic Features of BC

Our living biobank of BC PDOs was established in Figure S1. In detail, one to two regions were collected per specimen when feasible (Figure 1A). Among 83 tumor regions obtained from 65 breast cancer patients, 68 PDO cultures from 50 patients were successfully established, corresponding to a region‐level establishment rate of 81.9% and a patient‐level establishment rate of 76.9% (Table S1). These PDOs covered major molecular subtypes of breast cancer (Figure 1B). The success of PDO establishment was affected by factors such as the interval from surgical resection to sample processing, the percentage of viable cells, and the tissue digestion method. Successfully established PDOs derived from multi‐region sampling (n = 68; Figure S2A) were subsequently screened with four breast cancer therapeutics. Hematoxylin and eosin (H&E) staining revealed close histopathological similarity between organoids and their matched parental tissues (Figure 1C), and the PDOs displayed diverse morphologies (Figure S2B). Immunopathological marker analyses further confirmed that multi‐regional organoids accurately preserved breast cancer marker expression observed in the original tumors (Figure S2C).

**FIGURE 1:**
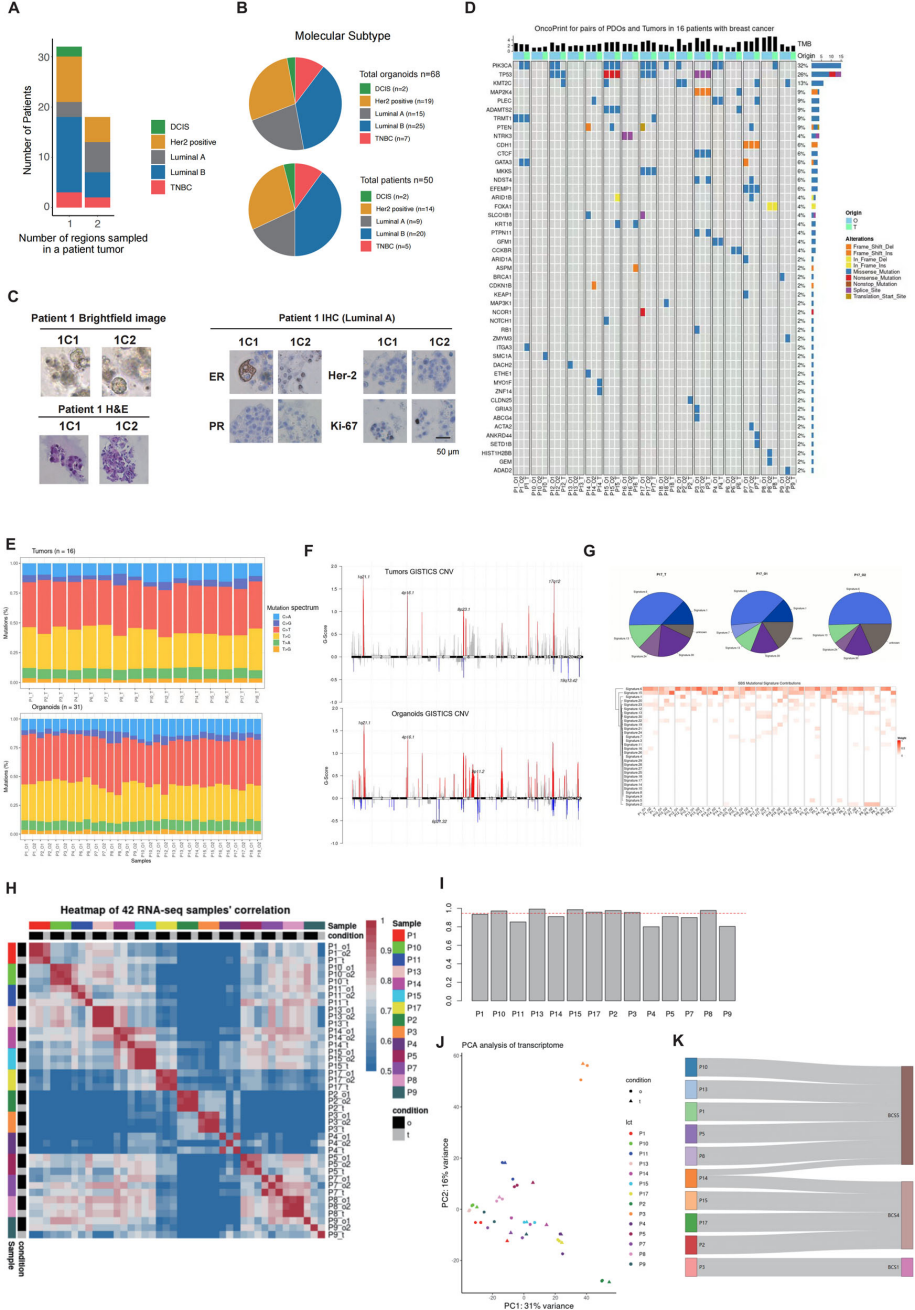
A biobank of multi‐region organoids of breast cancer. (A) Histogram summarizing the number of regions sampled from 50 patients, with colors indicating the histological subtypes of 48 patients with Invasive Ductal Carcinoma (IDC) Breast Cancer and two patients with Ductal Carcinoma In Situ (DCIS) Breast Cancer, respectively. (B) Pie charts illustrating the molecular subtypes of all 68 organoids shown in Figure S1, with numbers indicated in the brackets. (C) Brightfield microscopy images, H&E and IHC staining of two tumor regions from a breast cancer patient P1, and H&E, and IHC of the corresponding organoids. Breast cancer molecular subtype markers (ER, PR, Her‐2, and Ki‐67) were assayed by IHC. Scale bars indicate 50 µm. (D) Mutation landscape of breast cancer genes in patient‐derived organoids (PDOs) and tumor lesions. (E) Mutation spectra of tumors and PDOs for each sample. (F) Significant copy number variations (CNVs) in tumors and PDOs organized by chromosomal position, with key cytobands labeled (e.g., *HER2* locus: chr17q12). (G) Mutational signatures in tumors and PDOs. Pie plot shows the frequency of signatures detected in patient P17 (top). Heatmap of mutational signatures depicts the ubiquitous DNA damage repair related signature 6 in all samples (bottom). (H) Heatmap showing correlation between tumor tissue and paired organoids transcriptome. Samples from a same patient were grouped together. Rows represent organoids and columns represent tissues. (I) Bar plot demonstrating the average correlation between tumors and PDOs in each patient, with red dashed line marks the median level. (J) PCA analysis of transcriptomes, tumors and PDOs are plotted in different shapes and colored by the corresponding lineage. (K) Sankey plot demonstrating concordant molecular subtypes between parental tumors and matched PDOs based on transcriptomic profiling.

To determine whether the biobank adequately represented both inter‐ and intra‐ tumor heterogeneity, we performed whole‐exome sequencing (WES) and RNA‐seq on 16 paired PDOs and corresponding tumor samples. These samples were randomly chosen from the initial 32 organoids and included all patients with multi‐region sampling. The full cohort was profiled using RNA‐seq, yielding 28 PDOs with both transcriptomic and drug‐response data for biomarker development. Mutation burden was comparable between tumors and organoids. Consistent with prior breast cancer genomic studies [23, 24, 25, 26, 27, 28], recurrently mutated genes—including *PIK3CA* (32%), *TP53* (26%), and *KMT2C* (13%)—were frequently observed in the biobank (Figure 1D). Mutational spectra (Figure 1E) and marked copy number alteration (CNA) changes (e.g., 1q21.1, 4p16.1, 6p21.32, and 17q12; Figure 1F) were highly concordant between tumors and corresponding organoids. Mutational signature analysis revealed a dominant contribution of DNA mismatch repair–related signature 6 in patient P17, which was consistently detected across all samples from both tumors and PDOs (Figure 1G).

Transcriptomic analyses demonstrated strong associations between paired tumors and organoids (*n* = 16 pairs) (Figure 1H), using a published analytical framework [29]. A median of 94.5% concordance in cancer‐related mutations was observed between tumor tissues and their matched organoids (Figure 1I). Principal component analysis (PCA) of transcriptomes showed distinct clustering according to lineage (Figure 1J), and Sankey plot visualization further confirmed robust subtype conservation between patient tumors and derived PDOs (Figure 1K).

Collectively, these comprehensive analyses demonstrate that breast cancer PDOs retain their parental characteristics from histopathological‐, genomic‐, and/or transcriptomic‐level, supporting their utility for investigating both inter‐ and intra‐tumor heterogeneity.

### 2.2 Analysis of Important Clinical Agents Uncovers Variability Both Between and Within Tumors in How They Respond to Drugs and Pinpoints Gene Expression Patterns that can Predict This Sensitivity

We screened 68 PDOs derived from 50 patients with four clinically used breast cancer agents—doxorubicin, paclitaxel, cyclophosphamide, and docetaxel—revealing pronounced inter‐patient variability and distinct response patterns across individual models (Figure 2A). To model drug sensitivity, we leveraged transcriptomic profiles, as gene expression has been shown to predict patient responses to therapy [30, 31, 32]. PDOs with matched RNA‐seq and drug‐response data were included in the drug‐response modeling analysis. Doxorubicin response was defined using the median IC50 as the cutoff, and 411 genes showed significant correlations with doxorubicin sensitivity (Figure 2B).

**FIGURE 2:**
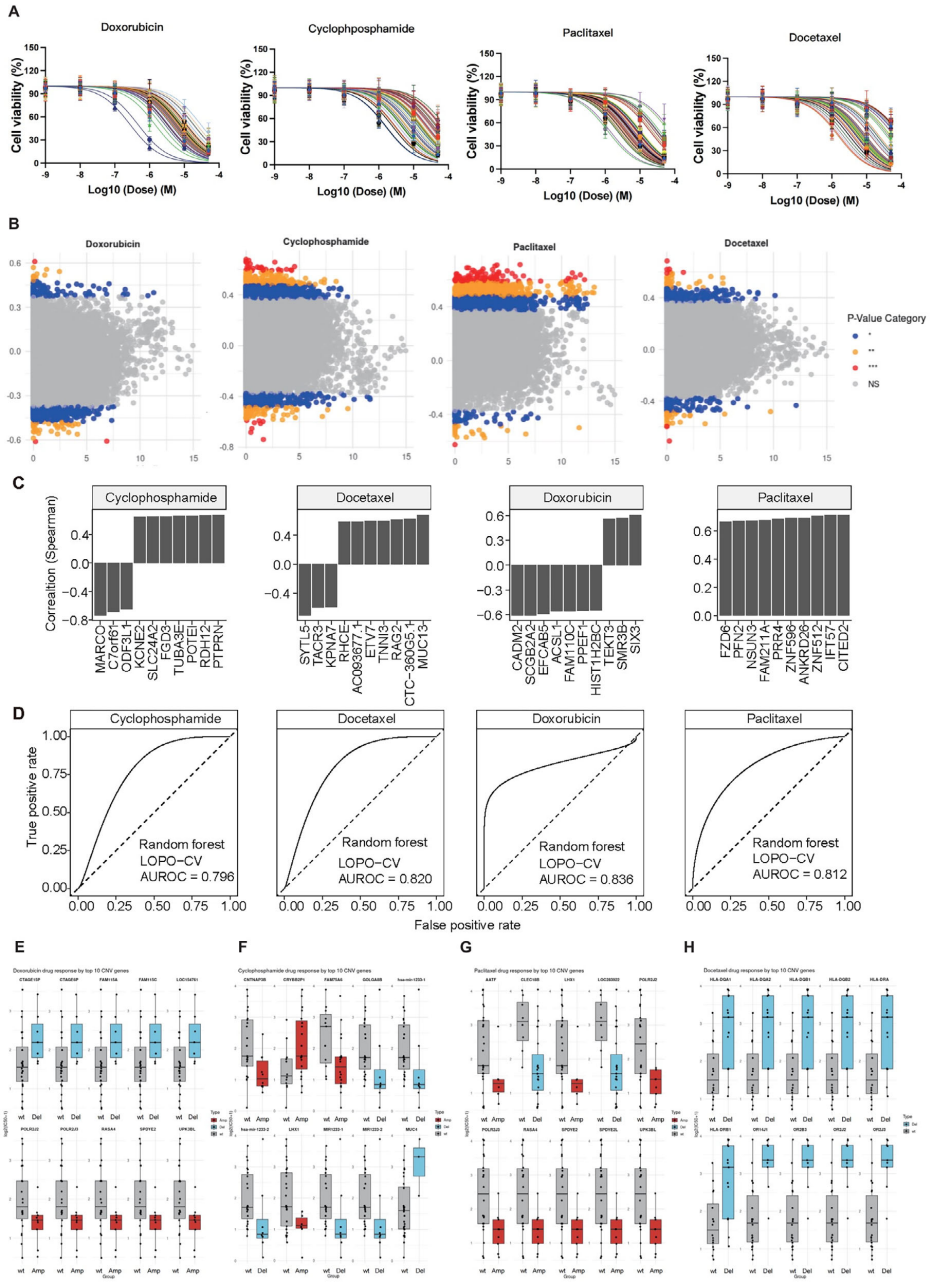
Drug screening and machine learning model. (A) Dose‐response curves of four breast cancer chemotherapy drugs for all screened 68 organoids (from 50 patients). Data represents relative cell viability values, with DMSO‐treated organoids used as control. Error bars represent means ± SD from at least triplicate experiments. (B) Scatter plot depicts correlation and expression of the correlated genes for each drug, colored by significance level. (C) Barplot of the selected top 10 genes for modeling response of four drugs. (D) ROC curves of random forest models for each drug using patient‐level leave‐one‐patient‐out cross‐validation (LOPO‐CV). (E–H) Box plot indicating association between genomic alterations in CNV‐associated genes and drug response to four therapeutic compounds, with color‐coding denoting specific alteration types.

Using a machine‐learning framework, we derived a 10‐gene expression signature associated with doxorubicin response, including ACSL1 (Figure 2C). To minimize potential information leakage caused by multi‐regional PDOs from the same patient, model performance was evaluated using patient‐level leave‐one‐patient‐out cross‐validation (LOPO‐CV), in which all regions from the same patient were held out together during validation. Under this more stringent validation strategy, the best‐performing random forest model achieved an AUROC of 0.836 for doxorubicin response prediction (Figure 2D, Figure S3C). Applying the same analytical framework to paclitaxel, cyclophosphamide, and docetaxel yielded patient‐level LOPO‐CV AUROCs of 0.812, 0.796, and 0.820, respectively (Figure 2D, Figure S3A,B,D). These results suggest that transcriptome‐based signatures retain predictive potential under patient‐level validation, although the relatively small cohort size supports interpreting these signatures as exploratory candidates requiring further validation. Analysis of the top 10 copy number variation (CNV)‐associated genes further revealed substantial heterogeneity in their relationships with drug sensitivity (Figure 2E–H).

Together, these data extend our prior findings and suggest that FAK–ACSL1 signaling might promote breast cancer progression by coordinating tumor–microenvironment interactions.

### 2.3 ACSL1 Mediates Doxorubicin Resistance

We selected doxorubicin, a widely studied drug, for further in‐depth investigation Elucidating mechanisms of doxorubicin resistance is critical for developing rational combination therapies. Doxorubicin induces apoptosis through topoisomerase II inhibition, DNA intercalation, and oxidative damage, yet its resistance mechanisms in breast cancer organoids remain incompletely understood.

We performed a protein–protein interaction (PPI) analysis (Figure 3A) using doxorubicin‐sensitivity genes to identify resistance‐related genes*. ACSL1* emerged as a central hub in this network and was among the 10 predictive signature genes, prompting further investigation. To establish its tumor‐specific environment, we developed a high resolution single‐cell atlas of the breast tumor microenvironment by combining scRNA‐seq data from 119 primary tumor biopsies collected from 88 patients, spanning 8 separate datasets (Figure 3B and Table S2) [33, 34, 35, 36, 37, 38, 39, 40]. After rigorous quality control to remove low‐quality cells and doublets, 236 363 cells spanning all clinical subtypes were integrated, with minimal batch effects observed. Cell types were annotated through typical markers and SingleR (Figure 3C). UMAP revealed conserved immune and stromal architectures across classes, whereas epithelial cells segregated consistently with prior reports, along groups [36, 38]. Single‐cell CNV analysis reliably distinguished malignant from normal epithelial populations (Figure 3D), providing a robust framework for microenvironmental characterization.

**FIGURE 3:**
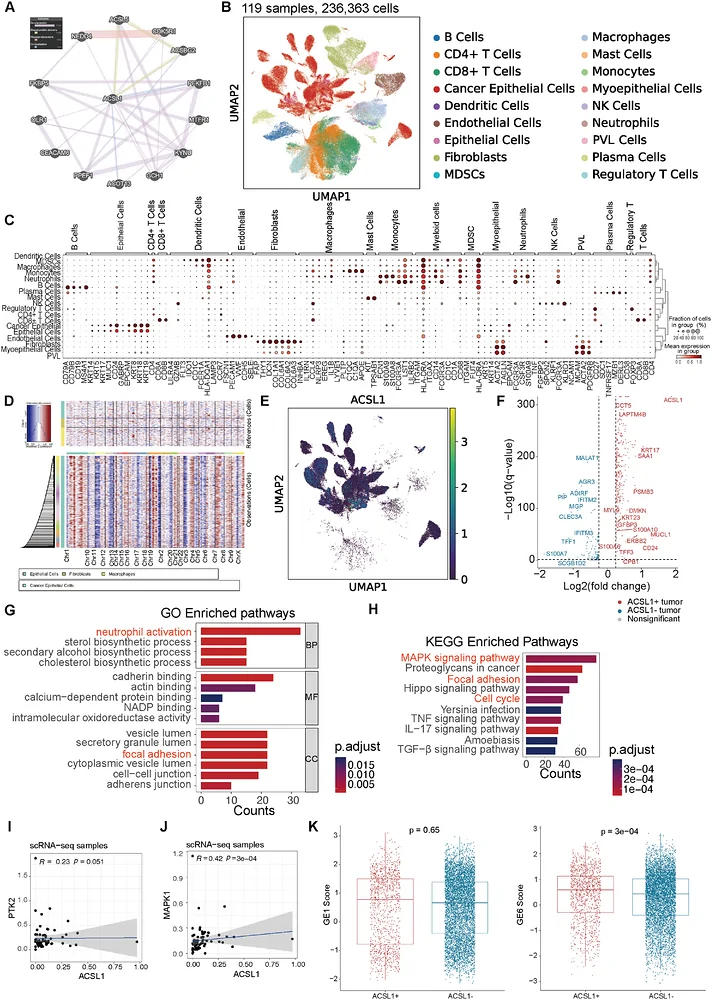
ACSL1 related pathways at single‐cell level. (A) Network based on top correlated genes with doxorubicin responses and curated interaction databases. (B) Uniform manifold approximation (UMAP) plot of 236,363 cells across 119 samples from 88 patients analyzed by scRNA‐seq. (C) Dot plot for expression of marker genes of different cell types. The color represents scaled average expression of marker genes in each cell type, and the size indicates the proportion of cells expressing marker genes. (D) Heatmap showing the inferred CNV profiles in malignant cells and their comparison with non‐malignant cells. (E) UMAP plot showing the expression levels of *ACSL1*, projected onto the same tumor cell type map as shown in B. (F) Differentially expressed genes of tumor cells with high (red) and low (blue) *ACSL1* abundances, stratified by the median level. Each red or blue point indicates a significant gene with BH‐adjusted *P* < 0.05 and |log2 (fold change) | > 0.25. (G, H) Gene ontology (GO) (G) and Kyoto Encyclopedia of Genes and Genomes (KEGG) (H) enrichment analysis showing pathways enriched in up‐regulated genes in *ACSL1*+ tumor cells compared to *ACSL1*‐ tumor cells. The number of genes identified in a pathway is proportional to the length of a bar, while statistical significance of enrichment (BH‐adjusted p value) is indicated by bar color. (I) Scatter plot showing positive correlation between mRNA levels of *ACSL1* and FAK. (J) Scatter plot showing positive correlation between mRNA levels of *ACSL1* and ERK. (K) Comparison of the GE1 score (left panel) and GE6 score (right panel) between ACSL1+ and *ACSL1*‐ tumor cells.

Comparative expression analysis between *ACSL1* ^+^ and *ACSL1* ^−^ tumor cells (Figure 3E) identified 261 differentially expressed genes (DEGs) (*p* < 0.05, |logFC| > 0.25) (Figure 3F). Enrichment analyses using GO and KEGG indicated a significant overrepresentation of neutrophil activation, focal adhesion, cell cycle, and MAPK signaling pathways in *ACSL1* ^+^ cells (Figure 3G,H). Detailed pathway interrogation showed upregulation of *PTK2* and *MAPK1* in focal adhesion and MAPK pathways, respectively (Figure 3I,J). GE1 and GE6 scores, which predict resistance to natural killer (NK) cell–mediated cytotoxicity [41], were significantly higher in *ACSL1* ^+^ tumor cells (Figure 3K), suggesting a link between ACSL1 expression and immune evasion.

Analysis of paired tumor and para‐cancerous tissues revealed significantly higher *ACSL1* expression in tumor lesions (Figure 4A). Functionally, knockdown of *ACSL1* increased sensitivity to doxorubicin in three resistant organoid models (Figure 4B). Previous evidence indicated that FAK inhibition downregulates *ACSL1* in bovine mammary epithelial cells [42]. Consistently, PTK2 and FAK signaling levels positively correlated with *ACSL1* expression in PDOs (Figure 4C,D), implicating FAK as an upstream regulator. *FAK* expression was also elevated in tumor versus para‐cancerous tissues (Figure 4E). Pharmacologic inhibition of *FAK* with IN10018 reduced FAK phosphorylation and *ACSL1* expression (Figure 4F). Moreover, combined treatment with doxorubicin and IN10018 was more effective than doxorubicin alone in eliminating breast cancer organoids (Figure 4G) and exhibited synergistic activity (Figure 4H).

**FIGURE 4:**
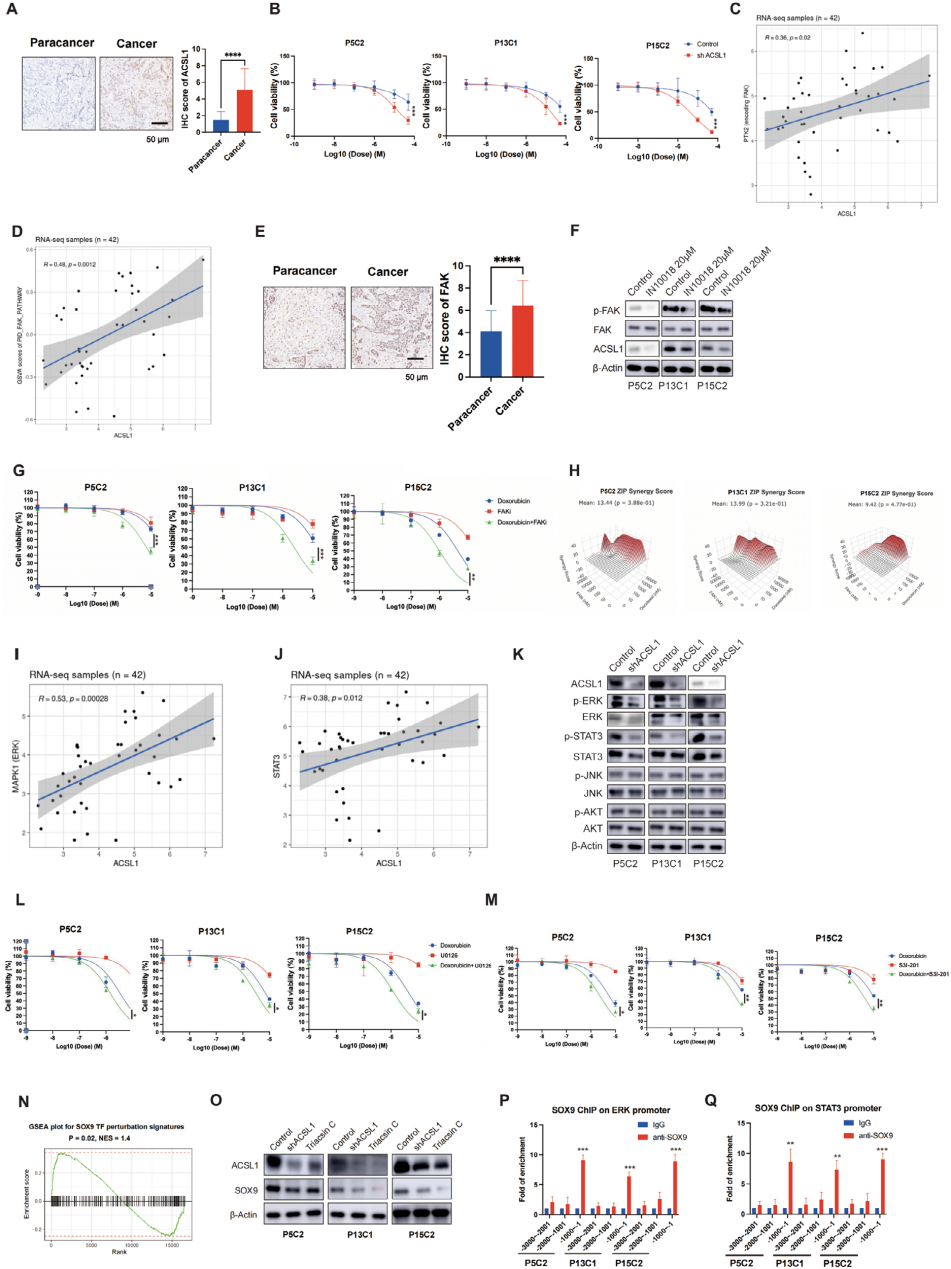
The FAK–ACSL1–ERK/STAT3 axis mediates doxorubicin resistance. (A) IHC staining of *ACSL1*(left panel) in two representative regions. IHC quantification of ACSL1 with all regions of tumor tissue and paracancer (right panel), showing a significant increase of ACSL1 expression in tumor tissue. (B) Dose response curves of doxorubicin upon ACSL1 knockdown (sh ACSL1) in three doxorubicin resistant organoids. Data are representative of at least three independent experiments. Error bars represent means ± SD. ***, *P* < 0.001. (C, D) scatter plots show expression of ACSL1 is significantly correlated with FAK (C) and FAK pathway activity (D). (E) IHC staining of FAK (left panel) in two representative regions. IHC quantification of FAK with all regions of tumor tissue and para‐cancer (right panel), showing a significant increase of FAK expression in tumor tissue. (F) Western blotting demonstrating a decrease of ACSL1 protein expression upon FAK inhibitor (FAKi) IN10018. (G) Dose‐response curves of doxorubicin plus IN10018 in three doxorubicin resistant organoids. Data are representative of at least three independent experiments. Error bars represent means ± SD. **, *P* < 0.01, ***, *P* < 0.001. (H) Synergy effect of doxorubicin plus IN10018 in three doxorubicin resistant organoids. (I‐J) Scatter plots show expression of ACSL1 is significantly correlated with ERK (I) and STAT3 (J). (K) Western blotting demonstrating decrease of p‐ERK and p‐STAT3 protein expression upon sh ACSL1. (L, M) Dose response curves of doxorubicin plus U0126 (ERK inhibitor) and S3I‐201 (STAT3 inhibitor) in three doxorubicin resistant organoids. Data are representative of at least three independent experiments. Error bars represent means ± SD. (N) GSEA analysis by using the SOX9 perturbation and ACSL1 DEGs. (O) Western blotting demonstrating a decrease of SOX9 protein expression upon shACSL1 or ACSL1inhibitor (5 µM, 24h treatment). (P, Q) qChIP analysis of the promoter of the ERK and STAT3 with antibodies against SOX9 protein proved that they were SOX9 direct target genes. *, *P* < 0.05, **, *P* < 0.01, ***, *P* < 0.001.

To define downstream signaling, we examined MAPK–ERK and STAT3 pathways, previously implicated in breast cancer drug resistance [43, 44], and found both positively correlated with *ACSL1* expression (Figure 4I,J). *ACSL1* knockdown reduced ERK and STAT3 phosphorylation (Figure 4K) without affecting other reported pathways [45]. Pharmacologic inhibition of *ERK* and *STAT3* using U0126 and S3I‐201 enhanced sensitivity to doxorubicin (Figure 4L,M). To elucidate how ACSL1 regulates these downstream signals, we analyzed differentially expressed transcription factors in high‐expressing *ACSL1* samples (Table S3) and identified SOX9 (Figure 4N), previously linked to breast cancer resistance [43], as correlated with ACSL1 expression. Accordingly, ACSL1 knockdown or treatment with the ACSL1 inhibitor triacsin C reduced *SOX9* expression (Figure 4O). Notably, triacsin C treatment was accompanied by reduced ACSL1 protein abundance in our system; however, whether this reflects altered protein stability, feedback regulation, or other indirect effects requires further investigation. Quantitative chromatin immunoprecipitation (qChIP) using anti‐SOX9 antibodies demonstrated direct binding of SOX9 to the *ERK* and *STAT3* promoters (Figure 4P,Q).

Collectively, these findings establish the FAK–ACSL1–ERK/STAT3 signaling axis as a key driver of doxorubicin resistance in breast cancer organoids.

### 2.4 FAK‐ACSL1 Signaling Promotes Doxorubicin Resistance in Breast Cancer Cell Lines and Xenograft Models

To assess whether ACSL1 universally mediates Doxorubicin resistance across breast cancer cell lines, we examined *ACSL1* expression following 24 h of Doxorubicin treatment in MDA‐MB‐231, SUM159, MCF‐7, and BT549 cells. Doxorubicin significantly increased *ACSL1* mRNA and protein (Figure 5A–C), whereas baseline *ACSL1* expression was markedly lower in the non‐malignant breast epithelial cell line MCF10A (Figure 5A,D). These findings indicate a conserved role for ACSL1 in Doxorubicin resistance across breast cancer models.

**FIGURE 5:**
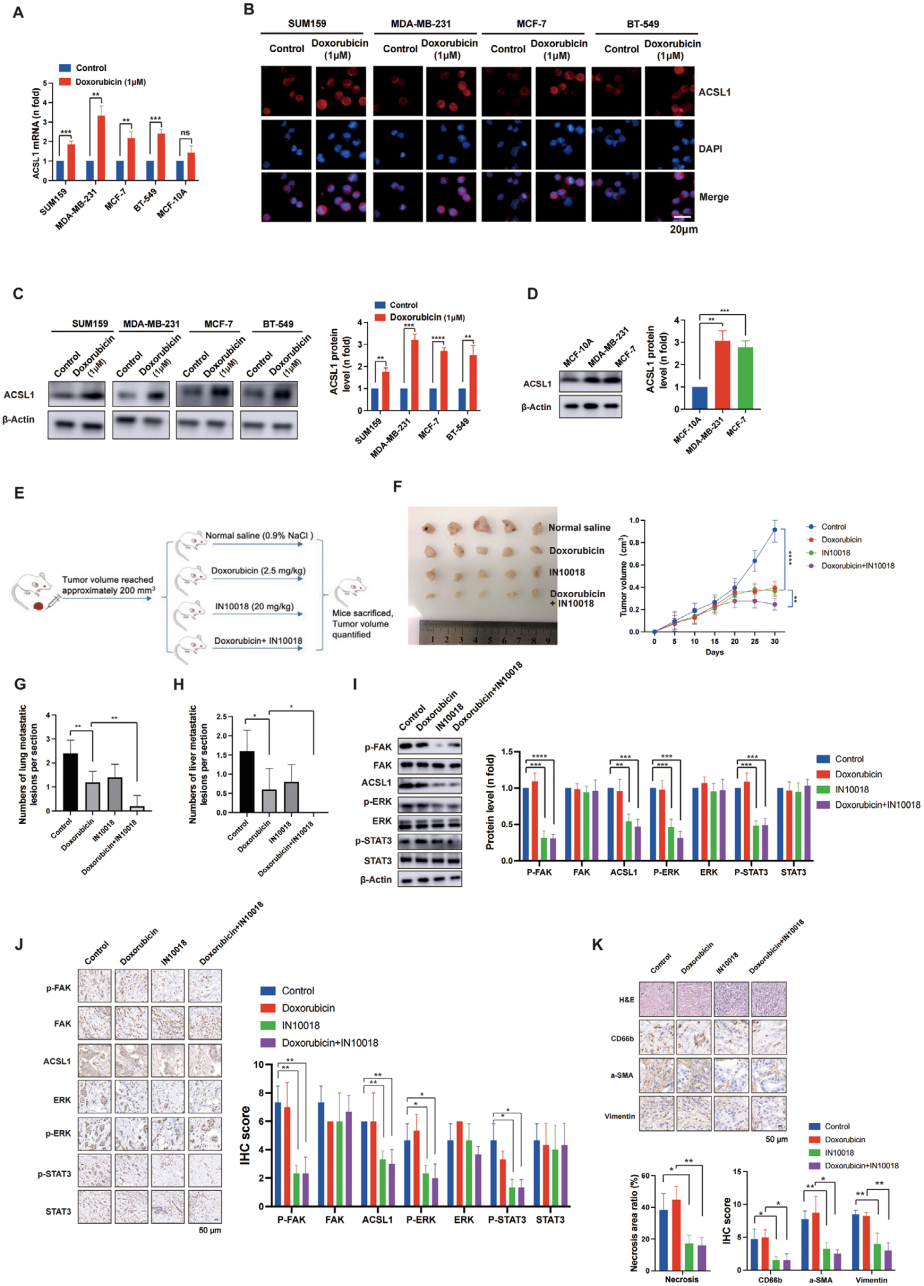
FAK inhibition enhances doxorubicin sensitivity in breast cancer. (A) qRT‐PCR demonstrated the increase of ACSL1 upon 24 h doxorubicin treatment in for breast cancer cell lines while the normal breast cell MCF‐10A not. (B) Immunofluorescence demonstrated the increase of ACSL1 upon 24 h doxorubicin treatment in for breast cancer cell lines. (C) Western blotting demonstrated the increase of ACSL1 upon 24 h doxorubicin treatment in for breast cancer cell lines (left) and quantified (right). (D) Western blotting showed expressions of ACSL1 in MCF‐10A, MCF‐7, and MDA‐MB‐231 cell lines (left) and quantified (right). (E) A proposed model of xenograft experiment. In brief, five million organoid‐derived cells were implanted subcutaneously of Balb/c nude mice. Dosing of the indicated drugs was initiated when tumor sizes reached 200 mm^3^ after 2 weeks. (F) Primary tumor size was measured (left) and quantified (right). (G, H) The numbers of metastatic lesions in lung (G) and liver (H) were quantified. (I, J) Western blotting (I) and IHC (J) illustrated the inhibition of FAK‐ACSL1 signaling. (K) Necrosis area ratio, α‐SMA and Vimentin levels in each group. Error bars represent means ± SD from triplicates. Data are representative of at least three independent experiments. *, *P* < 0.05, **, *P* < 0.01, ***, *P* < 0.001, ****, *P* < 0.0001.

To evaluate the contribution of ACSL1 signaling to chemoresistance in vivo, we tested the FAK inhibitor (FAKi) IN10018, which our team has previously demonstrated to be clinically effective (NCT05830539). Organoid‐derived cells (5 × 10^6^ cells) were implanted subcutaneously into the flank of female BALB/c nude mice (Figure 5E). When tumors reached approximately 200 mm^3^, mice were randomized into four treatment groups (*n*  =  5 per group): saline control (intraperitoneal injection three times weekly for two weeks), Doxorubicin (2.5 mg/kg intraperitoneally three times weekly for two weeks), IN10018 (20 mg/kg orally by gavage daily for two weeks), or combined Doxorubicin plus IN10018. Tumor volumes were monitored every two days and quantified every five days (Figure 5F). Combination treatment resulted in a marked reduction in primary tumor growth alongside lung and liver metastatic burden (Figure 5G,H), suggesting lower aggression. Western blotting (Figure 5I) and immunohistochemistry (IHC) (Figure 5J) of tumor tissues revealed reduced phosphorylation of ERK (p‐ERK) and STAT3 (p‐STAT3), concomitant with decreased p‐FAK and ACSL1 expression in the combination group. These data demonstrate that IN10018 sensitizes tumors to Doxorubicin in vivo. Moreover, IN10018 treatment was associated with reduced necrosis and reduced expression of cancer‐associated fibroblast (CAF) markers (α‐SMA and Vimentin), suggesting modulation of the tumor microenvironment (TME) (Figure 5K).

### 2.5 FAK–ACSL1 Signaling is Associated with Tumor Progression and Remodeling of the Tumor Microenvironment

To assess the clinical significance of ACSL1 signaling in the progression of BC, we measured the protein levels of components within the FAK–ACSL1 pathway in 139 breast‐cancer samples. These samples included 60 pairs of adjacent normal tissues, and the analysis was conducted using tissue microarrays (TMAs), following the methodology previously described [44]. Expression of FAK–ACSL1–associated molecules was significantly increased in tumor than in nearby breast tissues (Figure 6A). Notably, IHC staining demonstrated a positive association between ACSL1 expression and both TNM stage and histological grade (Figure 6B). Kaplan–Meier survival analyses using publicly available datasets (http://kmplot.com/analysis/), including GEO, EGA, and TCGA cohorts, revealed that high mRNA expression of the FAK–ACSL1 axis was associated with poorer overall survival (OS), relapse‐free survival (RFS), and distant metastasis‐free survival (DMFS) (Figure 6C).

**FIGURE 6:**
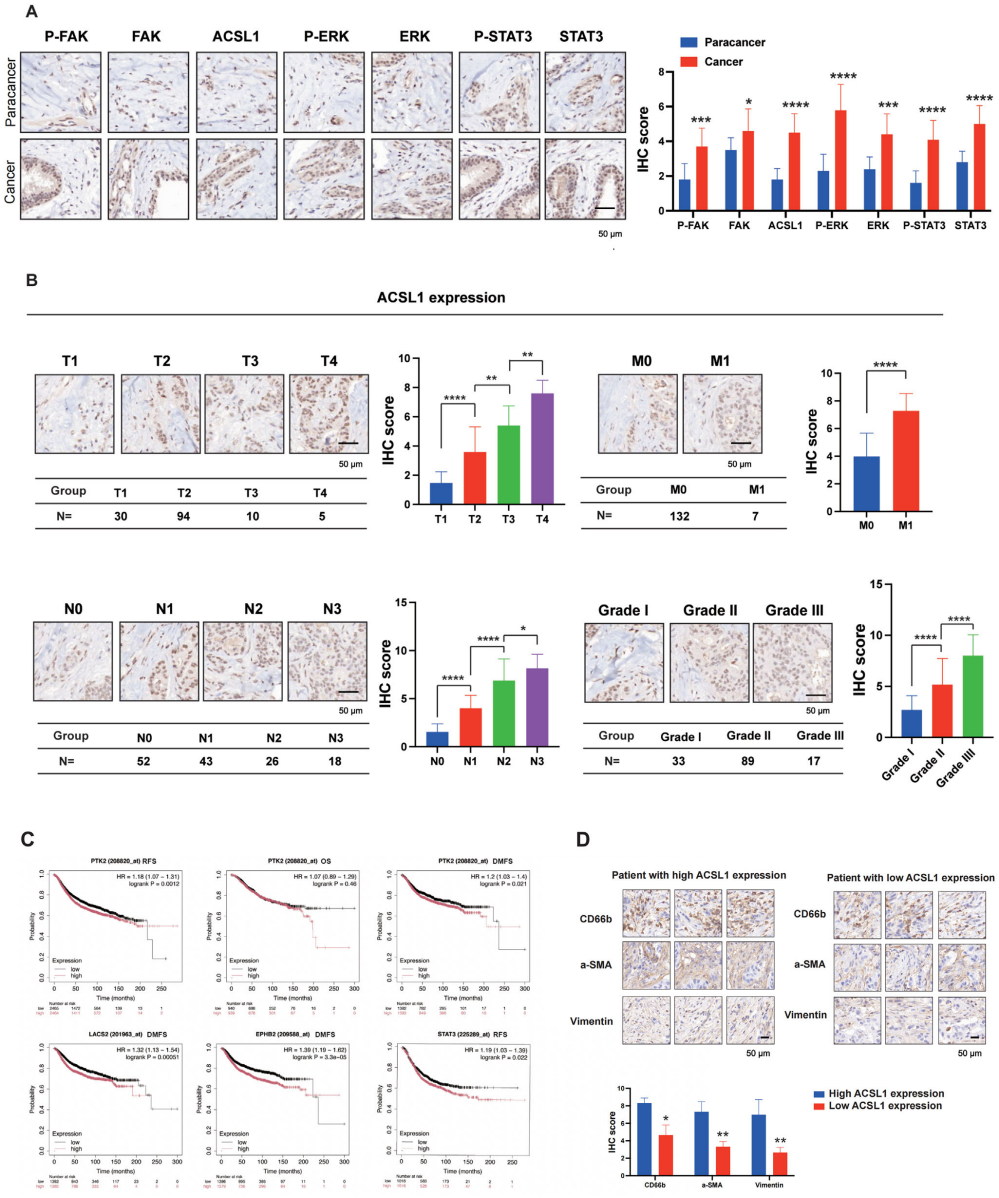
FAK‐ACSL1 axis is associated with clinical breast cancer progression. (A) IHC staining demonstrated that molecules involved in FAK‐ACSL1 axis were up‐regulated in breast cancer tissues (bottom) compared with their paired adjacent breast tissues (top, *n* = 60). (B) ACSL1 level was associated with breast cancer TNM stage and grade. (C) Kaplan–Meier survival analysis (http://kmplot.com/analysis/) showed the negative correlations between survival time of breast cancer patients and expressions of FAK‐ACSL1 axis. RFS relapse free survival, OS overall survival, DMFS distant metastasis free survival. (D) CD66b, α‐SMA, and Vimentin levels were associated with high ACSL1 expression. Error bars represent means± SD from triplicates. Data are representative of at least three independent experiments. *, *P* < 0.05, **, *P* < 0.01, ***, *P* < 0.001, ****, *P* < 0.0001.

Based on transcriptional signatures of *ACSL1* ^+^ cells, we investigated if tumor–TME interactions contribute to Doxorubicin resistance. Using CellPhoneDB analysis [46], we inferred intercellular communication networks underlying distinct tumor phenotypes. Compared with ACSL1^−^ tumor cells, ACSL1^+^ tumor cells displayed increased interactions with fibroblasts and endothelial cells, but reduced communication with B cells, plasma cells, and NK cells (Figure S4A). Consistent with pathway enrichment for neutrophil activation and focal adhesion (Figure 3G,H), *ACSL1* ^+^ tumor cells showed enriched CXCL14–CXCR4 ligand–receptor interactions with neutrophils, suggesting enhanced neutrophil recruitment. In addition, ligand–receptor pairs including TNFSF12–TNFRSF12A, LGALS9–P4HB, LRPAP1–SORT1, HBEGF–ERBB4/2, HBEGF–EGFR, CD93–IFNGR1, and AREG–EGFR were enriched between myeloid‐derived suppressor cells (MDSCs) and *ACSL1* ^+^ tumor cells (Figure S4B,C,E,H,I). Interactions between fibroblasts and ACSL1^+^ tumor cells were characterized by enrichment of WNT2–SFRP1, WNT2–FZD7/6/3/1–LRP6/5, IGF2–IGF2R, IGF2–IGF1R, and CNTN1–NOTCH2 (Figure S4D,E). Importantly, elevated IGF2R expression was associated with poor prognosis in the TCGA‐BRCA RNA‐seq cohort (Figures S4F,G). Consistent with these findings, high ACSL1 expression correlated with increased levels of CD66b, α‐SMA, and Vimentin, supporting a role for ACSL1 in remodeling the TME in patients (Figure 6D).

Together, these data extend our prior findings and demonstrate that FAK–ACSL1 signaling might promote breast cancer progression by coordinating tumor–microenvironment interactions.

## 3 Discussion

In this study, we established a multi‐regional breast cancer patient‐derived organoid (PDO) biobank that faithfully preserves the tissue and multi‐omics complexity of parental tumors. Our work extends recent advances in breast cancer organoid modeling, including the establishment of 95 organoids from 155 specimens by Sachs et al. [8] which captured tumor pathology and receptor status, as well as studies by Chen et al. [47] and Guillen et al. [48] demonstrating clinical relevance through drug response prediction and therapeutic guidance. Importantly, our multi‐regional sampling strategy robustly captured intra‐tumor heterogeneity, a defining feature of breast cancer that is frequently underrepresented in conventional models. The strong concordance in recurrent driver mutations, mutational signatures, copy number alterations, transcriptomic profiles, and molecular subtypes between PDOs and matched tumors underscores the reliability of this platform. Together, the high establishment efficiency and preservation of tumor diversity position this biobank as a powerful resource for investigating breast cancer biology and therapeutic vulnerability.

Using this resource, pharmacological profiling revealed marked inter‐patient and intra‐tumor heterogeneity in responses to standard breast cancer therapies. To create clinically useful tools from these results, we designed and tested multigene expression signatures for predicting several agents, including Doxorubicin, achieving high accuracy (AUROC > 0.9). These results highlight the feasibility of leveraging PDO transcriptomic data to stratify patients by therapeutic sensitivity and support the development of biomarker‐guided clinical trials.

Our findings also uncover a context‐dependent role for ACSL1 in therapy resistance. While prior work implicated *ACSL1* in resistance to hormone therapies such as tamoxifen in immunocompetent models [49], our study identifies Acyl‐CoA Synthetase Long Chain Family Member 1 (ACSL1) as an important mediator of chemotherapy resistance, specifically to Doxorubicin. ACSL1 emerged both as a core component of the predictive gene signature and as a central hub in the resistance‐associated PPI network. Functional experiments demonstrated that *ACSL1* knockdown sensitized resistant organoids to Doxorubicin. Integrative single‐cell RNA sequencing further revealed that *ACSL1* ^+^ tumor cells display distinct molecular programs enriched for focal adhesion, cell cycle, and MAPK signaling pathways and exhibit features associated with immune evasion. Consistent with prior studies showing that FAK phosphorylation drives intrinsic gemcitabine resistance in pancreatic cancer [50] and that FAK inhibition remodels the immunosuppressive microenvironment in PDAC [17], we identified a previously unrecognized FAK–ACSL1 signaling axis underlying chemoresistance. Specifically, FAK signaling positively regulated *ACSL1* expression, and pharmacologic FAK inhibition with IN10018 reduced ACSL1 levels and potentiated Doxorubicin efficacy. Downstream, ACSL1 activated ERK and STAT3 signaling, well‐established mediators of chemoresistance. ACSL1 knockdown reduced p‐ERK and p‐STAT3, and inhibition of these pathways restored Doxorubicin sensitivity, confirming their functional positioning downstream of ACSL1.

The clinical significance of the FAK–ACSL1 axis is strongly supported by our patient data. *ACSL1* levels were significantly higher in tumor samples compared to nearby normal breast tissue, and this increase was associated with more advanced TNM stages and larger tumors. High *ACSL1* mRNA levels were also associated with inferior OS, RFS, and DMFS. Concordantly, key components of the FAK–ACSL1 pathway, including FAK, p‐ERK, and p‐STAT3, were overexpressed in clinical breast cancer specimens. These findings establish the pathological relevance of this signaling cascade in disease progression and therapeutic resistance.

Beyond tumor‐intrinsic mechanisms, our data indicate that the FAK–ACSL1 axis contributes to resistance through remodeling of the tumor microenvironment. Computational inference revealed enhanced stromal interactions in *ACSL1* ^+^ tumor cells, including CXCL14–CXCR4 and WNT2–FZD signaling, and in vivo treatment with Doxorubicin plus IN10018 reduced CAF marker expression. Together, these findings suggest that tumor‐intrinsic FAK–ACSL1 signaling promotes chemoresistance not only through intracellular survival pathways but also by co‐opting stromal components to establish a protective microenvironment.

Notably, FAK/TIGIT co‐inhibition has been shown to reprogram the immunosuppressive tumor microenvironment and extend survival by inducing tertiary lymphoid structures (TLS) [51, 52], leading to FDA Fast‐Track Designation and the initiation of multiple multicenter trials (e.g., NCT05379946, NCT05982522, NCT05830539, and NCT06166836) evaluating FAK inhibitors in combination with Doxorubicin and/or immune checkpoint inhibitors (ICIs) across solid tumors. Our predictive signatures may provide a hypothesis‐generating framework for future biomarker‐guided studies of doxorubicin‐based combination strategies. However, larger independent cohorts and prospective validation will be required before these signatures can be used for patient stratification or clinical decision‐making.

The main finding of our study is the revelation of pharmacosensitive molecular subtyping of breast cancer patients based on an organoid biobank. Admittedly, due to the currently limited number of patients and organoids included, we did not strictly distinguish between different pathological subtypes of breast cancer when identifying drug resistance genes, but rather provided a broad generalization. This is the major limitation of our study. In future research, we will continue to expand the organoid biobank and drug sensitivity data to further analyze subtypespecific characteristics of breast cancer drug resistance.

In summary, we present a multi‐regional breast cancer PDO biobank that accurately captures tumor heterogeneity and enables functional interrogation of therapeutic resistance. Using this platform, we identify the FAK–ACSL1–ERK/STAT3 signaling axis as a central driver of Doxorubicin resistance. Briefly, ACSL1 acts as a valuable biomarker for prediction and highlight its potential as a target for therapeutic intervention. The predictive signatures and the FAK inhibitor combination strategy could provide promising clues for the clinical treatment of breast cancer.

## 4 Methods

### 4.1 Samples and Patients

BC tissue samples were collected from patients undergoing surgery at Puyang Oilfield General Hospital China. All samples were obtained with informed consent, following the principles of the Declaration of Helsinki, and received approval from the hospital's ethics committee (approval number 2024‐03‐0009‐E01). The clinical details of the patients are provided in Table S1.

Fresh tissues were transported on ice in F12/DMEM supplemented with 1% (v/v) penicillin/streptomycin (Gibco). Each specimen was subdivided into at least two portions for parallel histopathological evaluation and organoid culture; larger samples were additionally processed for DNA and RNA extraction for WES and RNA‐seq. PDO establishment was attempted using 83 tumor regions obtained from 65 breast cancer patients, and 68 tumor regions derived from 50 patients were successfully established, corresponding to a region‐level establishment rate of 81.9% and a patient‐level establishment rate of 76.9%.

### 4.2 Tissue Dissociation and Organoid Culture

Tissues were finely chopped into approximately 0.5 to 1 mm fragments and rinsed with PBS (Thermo Fisher) at 4°C. The samples underwent enzymatic dissociation with a tumor dissociation kit (Miltenyi Biotec), followed by a 0.5‐1.5h incubation (37°C). The digestion process was stopped by adding cold DMEM supplemented with 10% FBS. The cell mixture was then filtered through a 70‐micrometer nylon mesh and spun down at 500 × *g* for 5 min. To remove red blood cells, samples were treated with 1× RBC lysis buffer (Thermo Fisher Scientific) for 10 min (4°C).

Cells were suspended in 30% Matrigel with reduced growth factors (Corning) and then plated into either 24‐ or 48‐well plates made of ultralow‐attachment material, depending on the number of viable cells. After allowing the Matrigel to solidify for 30 min, pre‐warmed organoid isolation medium was added. This medium was modified from a previously published protocol [29] using ecombinant human Noggin at 25 ng/mL (PeproTech), Rspo‐1 at 500 ng/mL (Novoprotein), and Wnt3a at 100 ng/mL (SAB), replacing the original Noggin‐, Rspo‐1–, and Wnt3a‐conditioned media. Expansion medium (passage 2 onward) consisted of Advanced DMEM/F12 supplemented with 1% penicillin/streptomycin, 1% GlutaMAX, 10 mM HEPES, B27 supplement without vitamin A (1:50), N2 supplement (1:100), 1.25 mM N‐acetyl‐l‐cysteine (Sigma), 10 mM nicotinamide, 10 nM recombinant human (Leu15)‐gastrin I, 50 ng/mL recombinant human EGF, 100 ng/mL recombinant human FGF10, 25 ng/mL recombinant human HGF, 10 µM forskolin, 5 µM A83‐01, 10 µM Y‐27632 (Sigma), 25 ng/mL recombinant human *Noggin*, 500 ng/mL Rspo‐1, and 100 ng/mL *Wnt3a*. Media were refreshed every 3–4 days. Organoids were routinely passaged every 1 to 3 weeks, using either mechanical dissociation or treatment with 0.25% Trypsin–EDTA, at ratios ranging from 1:2 to 1:4. For freezing preservation, the organoids were broken down into single cells or small clusters and stored in a cryoprotective solution composed of 90% CS‐FBS and 10% DMSO, then kept at −80°C. The cryopreserved samples were successfully revived after storage periods extending up to approximately 18 months.

All tumor organoids (*n*  =  68) were independently evaluated by two pathologists using hematoxylin and eosin (H&E) staining.

### 4.3 Organoid‐Derived Tumor Xenografts

BALB/c nude mice, female, one month old, were sourced from the Center of Experimental Animals at Peking University First Hospital in Beijing, China, and kept in pathogen‐free environments. All procedures involving animals received approval from the Institutional Animal Care and Use Committee of Peking University First Hospital (Approval No. S2025086).

For subcutaneous xenografts, 5 × 10^6^ dissociated organoid cells for patient 13 (Her2 positive) were suspended in 10% Matrigel/90% F12/DMEM and then injected subcutaneously. When tumors reached ∼200 mm^3^, animals were randomized into 4 groups (5 each): saline control (intraperitoneal injection three times weekly for two weeks), Doxorubicin (2.5 mg/kg intraperitoneally three times weekly for two weeks), IN10018 (20 mg/kg by oral gavage daily for two weeks), or combined Doxorubicin plus IN10018. Tumor volumes were measured daily and quantified every 5 days using the formula: volume  =  ½ × length × width^2^. Statistical significance was assessed by ANOVA followed by Tukey's HSD post hoc test. All mice were euthanized five weeks after tumor inoculation.

### 4.4 Histology and Immunostaining

Tissues were fixed in 10% neutral‐buffered formalin (Sigma) for 24 h, while organoids were fixed for 0.5 h under the same conditions. Following fixation, samples were embedded in paraffin, sectioned into 5‐micrometer slices, and subjected to standard HE staining as well as immunohistochemistry (IHC). Primary antibodies against p‐FAK, FAK, ACSL1, ERK, p‐ERK, p‐STAT3, STAT3, CD66b, α‐SMA, and Vimentin (Abcam) were used at a 1:200 dilution. IHC scoring incorporated both staining intensity and extent. Intensity was graded as 0 (negative), 1 (weak), 2 (moderate), or 3 (strong). Extent was classified based on the percentage of positive tumor cells: 0 (<5%), 1 (5–25%), 2 (26–50%), 3 (51–75%), or 4 (>75%). Final scores were calculated as the product of intensity and extent, yielding a range of 0–12.

### 4.5 Whole‐Exome Sequencing and Data Preprocessing

Matched normal tissue from each patient served as the control for tumor organoid and tumor tissue samples. Genomic DNA was isolated using Qiagen's DNeasy and RNeasy kits, while exonic regions were targeted with Agilent SureSelectXT Human All Exon V6 probes. Sequencing was performed on the Illumina NovaSeq 6000 system, producing 150‐base pair paired‐end reads. The median sequencing depth was 233× in tumor samples, 234× in patient‐derived organoids, and 340× in the matched normal tissues.

Quality assessment of raw sequencing reads was performed with FastQC (v 0.11.9). Adapter sequences were removed using Cutadapt (v 2.5), followed by further trimming with Trimmomatic (v 0.39), applying paired‐end mode and a minimum read length of 36 bases. The resulting high‐quality reads were aligned to the UCSC human reference genome (hg19) with bwa‐mem2 [53] with default parameters. The BAM files underwent merging, sorting, and indexing processes using Samtools (v 1.10. PCR and optical duplicates were eliminated with GATK (v 4.1.2.0). Sequencing coverage statistics were computed with Samtools, focusing on exonic regions specified by the SureSelectAllExonV6r2 BED file. Median coverage was ×233 for tumor tissues (*n*  =  16), ×234 for PDOs (*n*  =  32), and ×340 for normal controls (*n * =  17).

### 4.6 Somatic Mutation Calling

Somatic single‐nucleotide variants, along with small insertions and deletions, were detected using Mutect2 (v 4.1.2.0) in paired tumor–normal mode, employing the normal tissue as a control. The variant identification involved filtering based on these criteria: a minimum of 10× coverage of the wild‐type allele in the normal sample with no reads supporting the mutant allele; 10× overall coverage in the tumor sample with a minimum of three reads supporting the mutation; and the removal of reads with an average base quality score below 20 at the variant position. Candidate variants were further filtered to retain only those with population frequencies <1% in ExAC, gnomAD_exome (v 2.1.1), and 1000 Genomes (August 2015). Variants were annotated using ANNOVAR. For comparisons of concordant non‐silent mutations in cancer‐related genes between tissue–organoid pairs and among multi‐region samples, inconsistent variants were manually reviewed to rescue potentially missed calls due to tumor purity, following a published strategy [29]. Tumor mutation burden was calculated as the total number of non‐silent mutations divided by the total exonic bases (38.3 Mb; Agilent V6).

### 4.7 Copy Number Analysis

Copy number alterations (CNAs) were detected through the use of CNVkit with its default settings. To pinpoint recurrent CNAs of significance, GISTIC2.0 was employed [54] with a q‐value threshold of < 0.25. These significant CNAs served as a basis for comparing the genomic profiles of organoids with those of corresponding tumor tissues.

### 4.8 RNA‐seq and Quantification

Total RNA was isolated employing DNeasy and RNeasy kits from Qiagen, followed by enrichment using magnetic beads conjugated with poly‐T oligos. The libraries were sequenced on the Illumina NovaSeq 6000 system, producing 150‐base pair paired‐end reads. Quality assessment of raw reads was performed with FastQC (v 0.11.9), Cutadapt (v 2.5, for Illumina universal adapters), and Trimmomatic (v 0.39, PE mode, minimum length 36). Reads were mapped to the human reference genome (hg19) using STAR (v 2.7.3a) with default settings. Gene counts were generated via HTSeq‐count using GENCODE annotations, and transcripts per million (TPM) values were calculated with RSEM [55]. Unless indicated otherwise, mRNA expression levels are presented as log2(TPM + 1). Correlation heatmaps comparing tissue samples and corresponding organoids were produced following the reported methods [20].

### 4.9 Drug Screening

Four clinically relevant anti‐cancer agents were used for pharmacological profiling. All compounds were dissolved in DMSO and stored at −80°C. Organoids were dissociated into clusters (<70 µm); samples resistant to mechanical or enzymatic dissociation were filtered through a 70 µm strainer to remove large aggregates. Organoids were plated in ultralow‐attachment 96‐ or 384‐well plates at ∼100 organoids per 100 µL in 5% Matrigel/culture medium. A 7‐point, 5–10‐fold serial dilution of each drug was applied (50, 10, 1, 0.1, 0.01, 0.001, and 0 µm; control), with a maximum DMSO concentration of 1%. After 6 days of treatment, cell viability was measured using the CCK‐8 assay according to the manufacturer's instructions and normalized to the control. IC50 and AUC values were calculated in GraphPad Prism 9 using nonlinear regression with the log(inhibitor) versus normalized response model [56]. Each concentration was tested in at least triplicate.

### 4.10 DEGs Enrichment

DEGs between the drug‐sensitive and drug‐resistant groups were identified using DESeq2 (v1.38.3), applying criteria of an absolute fold change greater than 2 and an adjusted *P*‐value less than 0.05. The resulting ranked lists of DEGs were analyzed for enrichment in Gene Ontology biological processes (GO_BP) utilizing MSigDB (v7.5.1), with significance threshold set at q‐values below 0.25. Further pathway enrichment analyses were performed using ClusterProfiler (v4.6.2).

### 4.11 Machine Learning Model of Drug Response

To predict drug response from transcriptomic data, we implemented a transcriptome‐based modeling framework for four agents: doxorubicin, paclitaxel, cyclophosphamide, and docetaxel. PDOs with matched RNA‐seq and drug‐response data were included in the modeling analysis. For each drug, PDOs were classified as responsive or nonresponsive using the median IC50 value as the cutoff, with PDOs below the median IC50 classified as responsive and those above the median IC50 classified as nonresponsive.

For analyses involving multi‐regional PDOs, regional PDO models were retained as distinct biological samples when assessing intra‐tumor heterogeneity. For predictive modeling, patient‐level leave‐one‐patient‐out cross‐validation was used to avoid information leakage. All PDOs derived from the same patient were assigned to the same fold, ensuring that regional PDOs from one patient were not simultaneously included in the training and validation sets.

Candidate genes were ranked according to their correlation with drug response in the organoid cohort, using incremental correlation thresholds with a step size of 0.01 and constraining candidate gene sets to 100–1,000 features. To reduce model complexity and improve interpretability given the limited sample size, the top 10 candidate genes for each drug were retained for final model training.

To address the potential non‐independence of multi‐regional PDOs derived from the same patient, we further evaluated the prediction models using patient‐level leave‐one‐patient‐out cross‐validation (LOPO‐CV). In each LOPO‐CV fold, all PDO regions derived from one patient were held out together as the validation set, while PDOs from the remaining patients were used for model training. Thus, regional PDOs from the same patient were never split between training and validation sets, avoiding patient‐level information leakage.

Several machine‐learning algorithms, including LASSO, ridge regression, elastic net, XGBoost, and random forest, were evaluated using the candidate signature genes. The best‐performing model for each drug was selected based on patient‐level LOPO‐CV AUROC. Model performance was assessed using receiver operating characteristic (ROC) curves and area under the ROC curve (AUROC). Feature importance scores were extracted from the best‐performing random forest model for each drug to improve model interpretability and identify the top‐ranked predictive genes (Figure S3A–D). Modeling analyses were performed in R using the glmnet (v4.1‐3), randomForest (v4.7.1.2), pROC (v1.18.5), and ROCR (v1.0‐11) packages. Details of patients and organoids included in transcriptomic and drug sensitivity analyses were listed in Table S5.

### 4.12 Processing and Integration of Single‐Cell RNA‐seq Datasets

We integrated 119 treatment‐naïve primary breast tumor specimens from 88 female patients across eight publicly available datasets using BBKNN (v1.5.1) for batch correction [57]. All additional preprocessing steps followed previously established protocols [41].

### 4.13 Cell Type Annotation and Clustering

Cell identities were assigned using canonical markers from the literature [41, 58, 59] and the SingleR (v 2.4.1) R package [60]. Copy number variation analysis based on filtered expression matrices was performed using infercnv (v1.2.1) [61].

### 4.14 Cell–Cell Communication Analysis

Potential intercellular communication networks among cell types in the scRNA‐seq datasets were inferred using CellPhoneDB (v5) [46].

### 4.15 Cell Lines and Cell Culture

Human breast cancer cell lines SUM159, MDA‐MB‐231, and BT549, along with the normal breast epithelial cell line MCF10A, were sourced from the American Type Culture Collection (ATCC) in Manassas, VA, USA. The MCF‐7 cell line was obtained from the Cell Resource Center at the Institute of Basic Medical Sciences, Chinese Academy of Medical Sciences in Beijing, China. The culture conditions followed the previously described protocols [43]. All cell cultures were incubated at 37°C within a humidified chamber containing 5% CO_2_. The identity of each cell line was validated through short tandem repeat (STR) analysis, and contamination was ruled out by testing for mycoplasma using a PCR‐based detection kit from Sigma, located in St. Louis, Missouri.

### 4.16 Plasmids and Transfection

Plasmids encoding shNC and shACSL1 were purchased from Mailgene Biosciences (Beijing, China). Organoid‐derived cells were transfected using Lipofectamine 3000 (Invitrogen) according to established protocols [62]. Stable transfectants expressing the neomycin phosphotransferase (neo) gene were selected by culturing in organoid medium supplemented with 400 µg/mL G418 (GIBCO) for 14 days.

### 4.17 ChIP and qPCR

ChIP assays were performed following as previously outlined [63, 64]. See Table S4 for qPCR primers.

## Author Contributions

H.Y., H.L., H.X. and J.C. supervised all of the research. H.Y. and H.X. established the organoid biobank. J.C. and J.Y. performed genomic analyses, bulk and single‐cell transcriptomic analyses, and implemented the machine learning models. Z.Q. H.X. and H.Y. collected clinical samples and clinical information. H.Y., B.Z., and M.C. performed experiments. J.Y. and J.C. designed the machine learning models and interpreted the analysis results. B.Z., Z, W. and P.W. provided technical guidance of experiments. H.Y. and J.Y. wrote the manuscript. H.Y., J.Y., H.X., J.C. and L.W. revised the manuscript. All authors commented on the manuscript.

## Funding

This work was supported by Tianjin Health Research Project (Grant No.TJWJ2025QN027), Tianjin Key Medical Discipline Construction Project (Grant No.TJYXZDXK‐3‐003A), the National Natural Science Foundation of China (No. 82573723 and 82003187), National High Level Hospital Clinical Research Funding (Scientific Research Fund of Peking University First Hospital, No. 2024XTZ10, “Star of Outlook” Scientific Research Project of Peking University First Hospital, No.2024XW02), the State Key Laboratory of Natural and Biomimetic Drugs, National Natural Science Cultivation Fund of Peking University First Hospital (No. 2024PY02), Clinical Medicine Plus X – Young Scholars Project of Peking University, the Fundamental Research Funds for the Central Universities (No. PKU2026PKULCXQ015).

## Conflicts of Interest

The authors declare no conflicts of interest.

## Supporting information




**Supporting File 1**: advs76541‐sup‐0001‐SuppMat.pdf.

**Supporting File 2**: advs76541‐sup‐0002‐Table_S1 v2.xlsx.

**Supporting File 3**: advs76541‐sup‐0003‐Table_S2 v2.xlsx.

**Supporting File 4**: advs76541‐sup‐0004‐Table_S3 v2.xlsx.

**Supporting File 5**: advs76541‐sup‐0005‐Table_S4 v2.xlsx.

**Supporting File 6**: advs76541‐sup‐0006‐Table_S5 v2.xlsx.

## Data Availability

The data that supports the findings of this study are available in the supplementary material of this article.
