# Supplementary material for: Multi‐regional Organoid Biobank Reveals FAK‐ACSL1‐Driven Doxorubicin‐Resistance and Predictive Biomarkers in Breast Cancer
Source: Adv Sci (Weinh). 2026 Jul 20;13(55):e76541. doi: 10.1002/advs.76541 (PMC13384037; doi:10.1002/advs.76541)
Supplement: Supplementary file 1 [file ADVS-13-e76541-s004.pdf]

Supplementary Figure 1

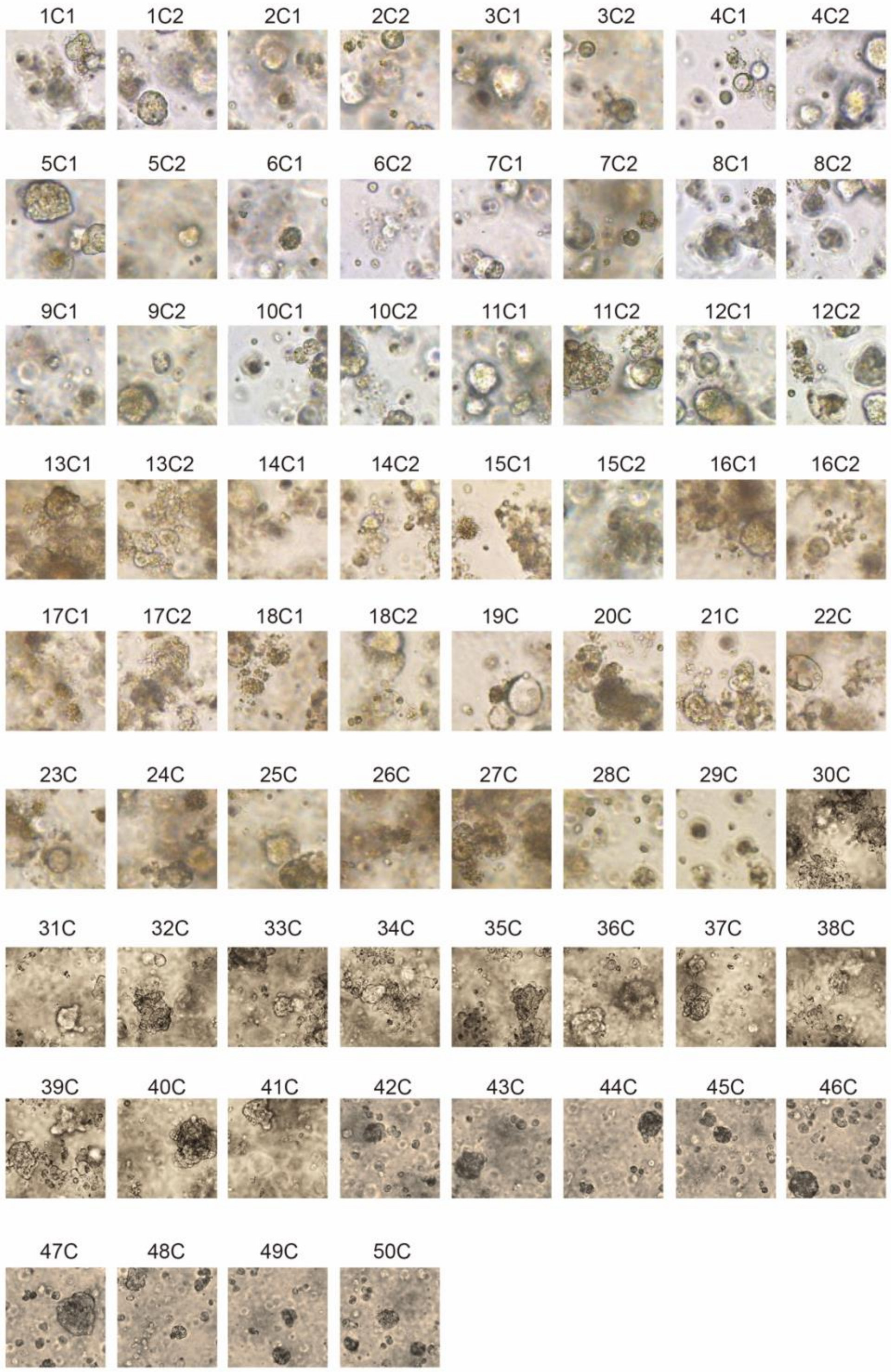

Figure S1 Brightfield microscopy images of 68 organoid lines derived from 50 patients with multiple-region sampling.

Supplementary Figure 2

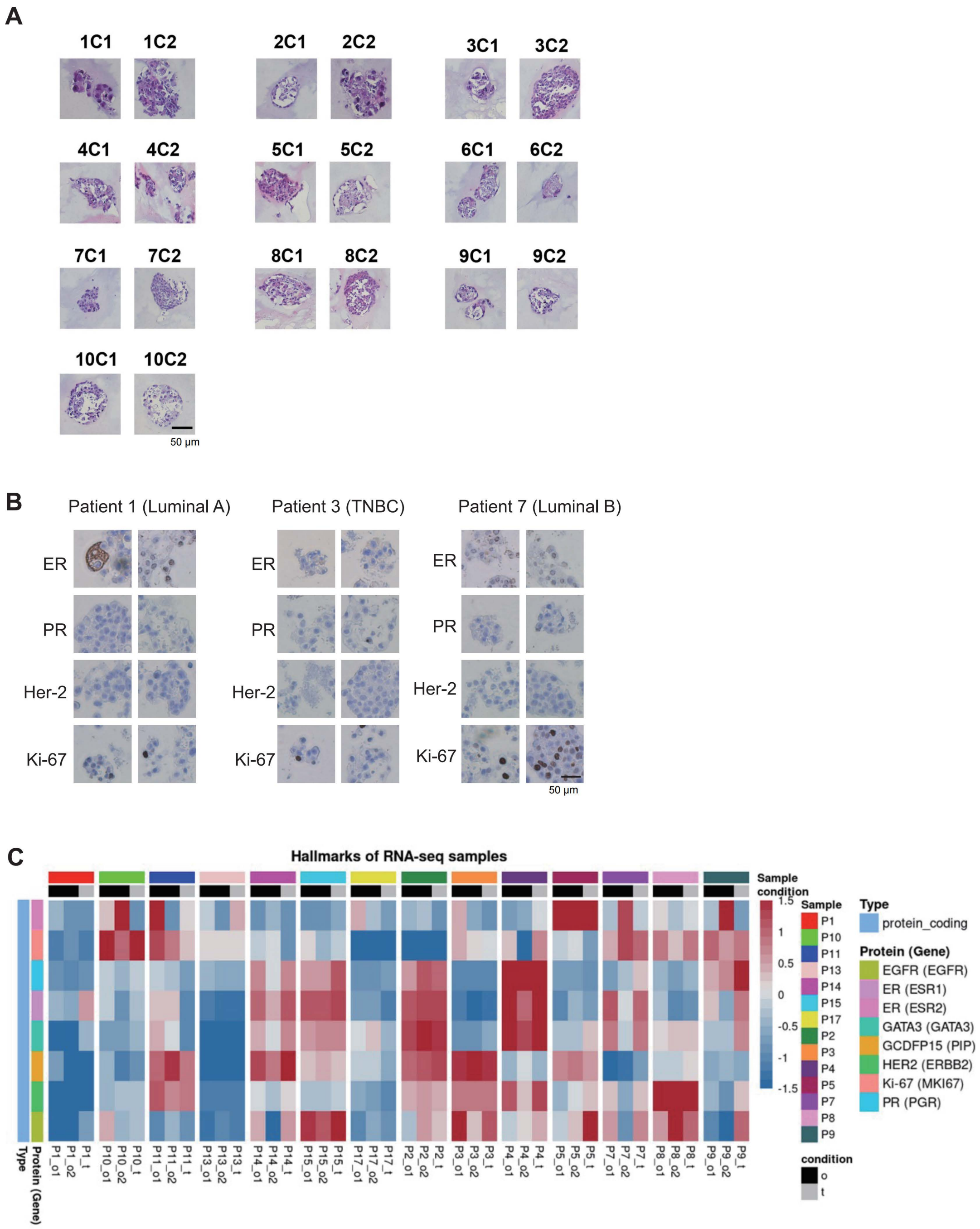

Figure S2 Multi-regional breast cancer organoids recapitulate the histological and molecular features of matched parental tumors.

A, H&E staining of representative multi-regional tumor tissue and the derived organoids.

B, IHC staining of breast cancer molecular subtype markers for representative multi-regional tumor tissue and the derived organoids.

C, Heatmap showing expression profiles of breast cancer molecular subtype markers in multi-regional tumor-derived organoids.

Supplementary Figure 3

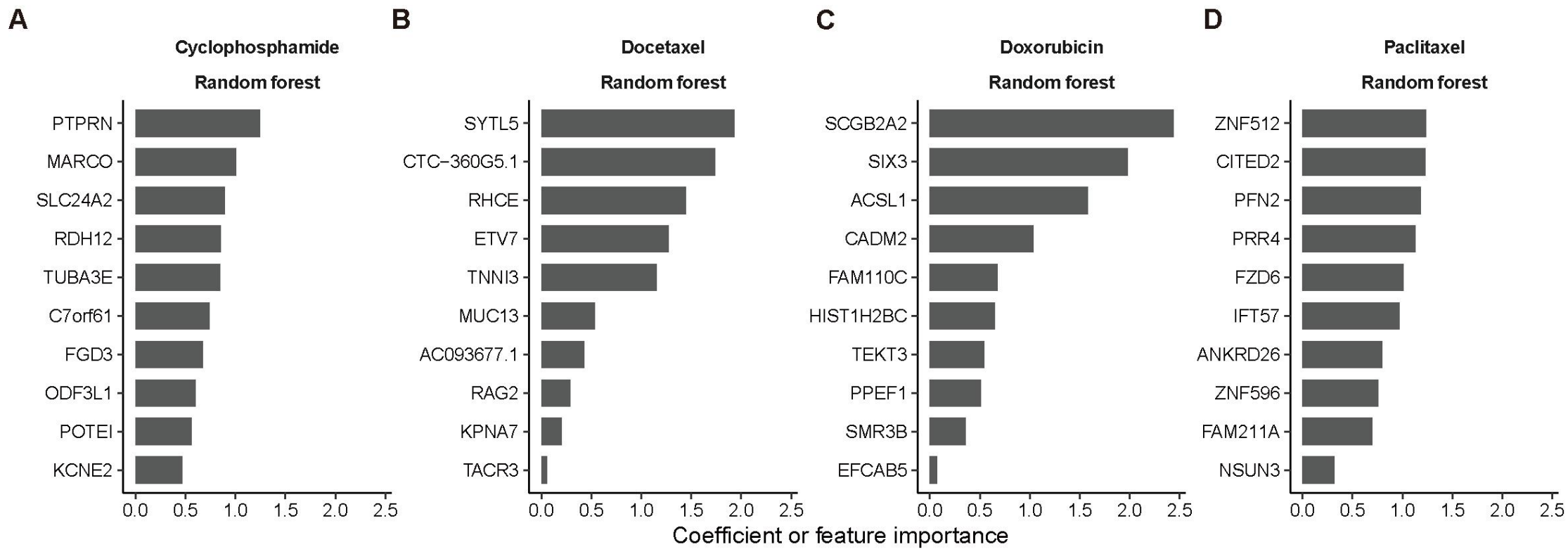

Figure S3 Feature importance of the best-performing random forest drug-response prediction models. A–D, Top-ranked predictive features from the best-performing random forest models for Cyclophosphamide (A), Docetaxel (B), Doxorubicin (C), and Paclitaxel (D) response prediction. Bars indicate random forest-derived feature importance scores, reflecting the relative contribution of each gene to model prediction.

Supplementary Figure 4

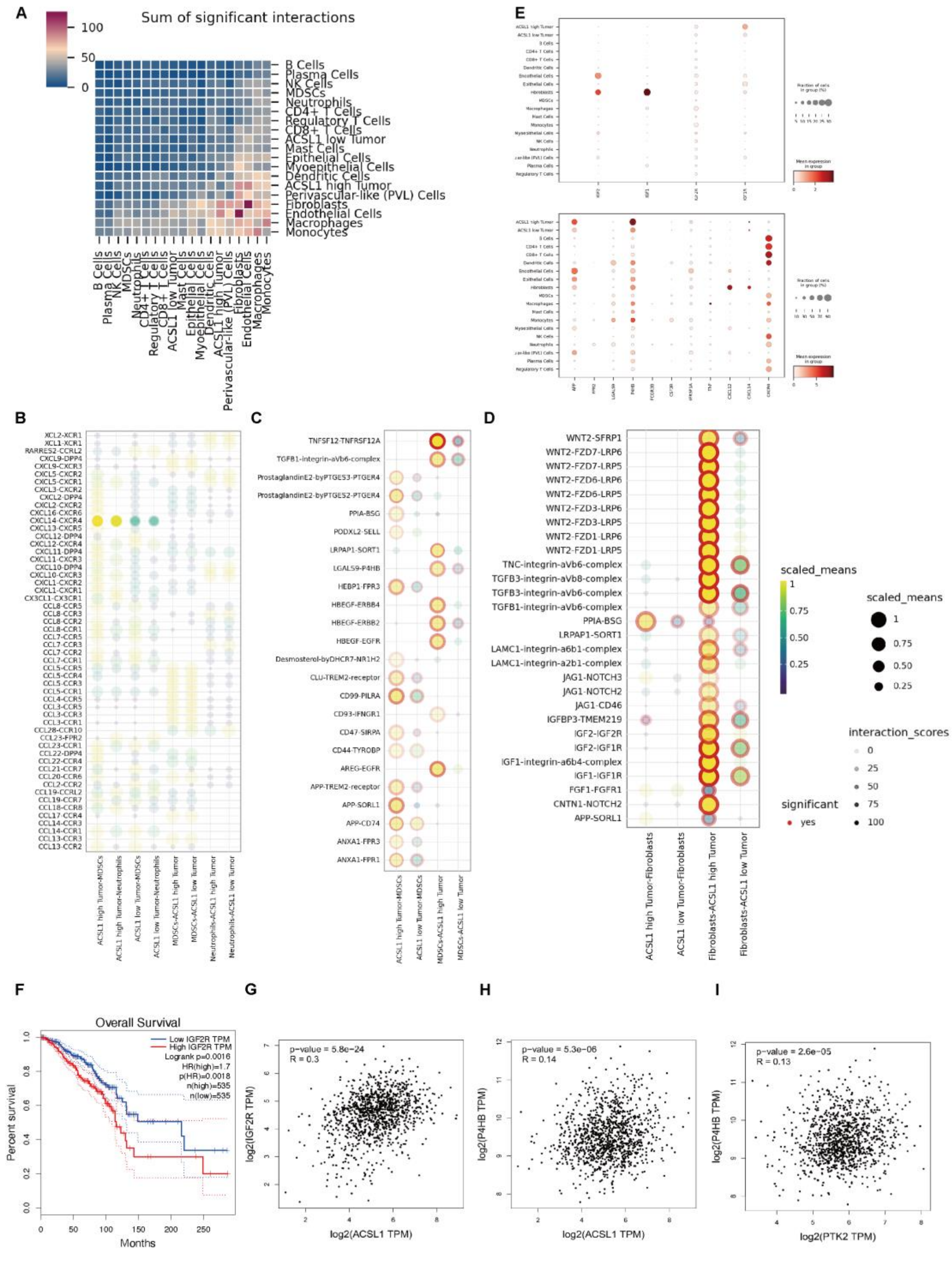

Figure S4 Intercellular communication and clinical relevance of the FAK–ACSL1 axis in the breast cancer tumor microenvironment.

A, Heatmap showing the overall strength of intercellular communications in ACSL1+ and ACSL1- tumor cells.

B, Dot heatmap showing the chemokine ligand-receptor pairs between ACSL1+/- tumor cells and neutrophils.

C, Dot heatmap showing the selected ligand-receptor pairs between neutrophils and ACSL1+/-tumor cells.

D, Dot heatmap showing the selected ligand-receptor pairs between ACSL1+/- tumor cells and fibroblasts.

E, The expression of specific ligands and receptors in tumor cells and TIME cells.

F, Kaplan–Meier survival analysis showed the negative correlations between survival time of breast cancer patients and expressions of FAK-ACSL1 axis.

G, Scatter plot showing positive correlation between mRNA levels of ACSL1 and IGF2R.

H, Scatter plot showing positive correlation between mRNA levels of ACSL1 and P4HB.

I, Scatter plot showing positive correlation between mRNA levels of PTK2 and P4HB.
